# Short-Term Postoperative Cognitive Dysfunction and Inflammatory Response in Patients Undergoing Cytoreductive Surgery and Hyperthermic Intraperitoneal Chemotherapy: A Pilot Study

**DOI:** 10.1155/2017/3605350

**Published:** 2017-11-09

**Authors:** Hui Yu, Rui Dong, Yayuan Lu, Xi Yang, Chang Chen, Zongze Zhang, Mian Peng

**Affiliations:** Department of Anesthesiology, Zhongnan Hospital of Wuhan University, 169 Donghu Road, Wuhan, Hubei, China

## Abstract

**Objectives:**

To assess the association between short-term postoperative cognitive dysfuction (POCD) and inflammtory response in patients undergoing cytoreductive surgery (CRS) and hyperthermic intraperitoneal chemotherapy (HIPEC).

**Design:**

A prospective cohort study.

**Setting:**

University medical centre.

**Participants:**

Fifty-one adult patients who had undergone CRS-HIPEC and twenty control participants.

**Measurements:**

The inflammatory marker levels in plasma and cognitive function were measured.

**Results:**

Twenty (39.2%, 20/51) patients developed POCD at 1 w after CRS-HIPEC. The patients with POCD had higher serum interleukin 1*β* (IL-1*β*), serum amyloid A (SAA), S100 calcium-binding protein *β* (S-100*β*), and high mobility group box-1 protein (HMGB-1) levels at 1 and 24 h postoperatively than patients without POCD. There was an association between POCD and the maximum IL-1*β* and S-100*β* concentrations in serum, which remained following adjustment for age and FBS.

**Conclusion:**

In this pilot study, perioperative inflammatory marker levels increase significantly after CRS-HIPEC in adult patients, and such elevations are associated with the development of short-term cognitive dysfunction after this complex surgery. These results suggested the need for a larger RCT to replicate and confirm these findings.

## 1. Introduction

Postoperative cognitive dysfunction (POCD) [1] is one of the most common postoperative complications in elderly patients and is associated with increased morbidity and mortality [2]. Advancing age, duration of anesthesia, and multiple surgeries have been implicated as the risk factors for POCD [2, 3]. However, despite the clinical importance of POCD, its neuropathogenesis remains largely unknown. Inflammation plays a crucial role in the development of POCD [4]. Surgery induces the tissue damage, which activates the peripheral innate immune system, leading to activation of the cytokine cascade and inflammatory mediator release [5]. The excessive systemic inflammation triggers the inflammatory process in the brain, which produces neurotoxic responses, affects neuronal function, and causes cognitive impairments. The proinflammatory cytokines, such as interleukin 1*β* (IL-1*β*) and tumor necrosis factor *α* (TNF-*α*), have been reported to play an important role in mediating surgery-induced systemic and central inflammation, ultimately resulting in cognitive decline [4, 6, 7].

The advent of the cytoreductive surgery (CRS) associated with hyperthermic intraperitoneal chemotherapy (HIPEC) in the early 1990s has dramatically changed the treatment of peritoneal surface malignancies. The combined treatment has been suggested as the standard of care for peritoneal and mesothelioma carcinomatosis related to ovarian and gastrointestinal cancers [8]. CRS consists of a complicated surgical procedure, which is associated with significant blood loss, fluid shifts, and prolonged duration of surgery [9]. Similarly, HIPEC involves the hemodynamic and metabolic perturbations [10] caused by the hyperthermic chemotherapy, which may contribute to an imbalance in cerebral tissue oxygen supply. As we know, the severity of the surgery and the incision size have an impact on the magnitude of postoperative inflammatory response [11, 12]. Additionally, the extent of surgery also contributes to the impaired cognitive function after surgery [13]. Therefore, it is possible that the combined treatments of CRS and HIPEC are related to severe systemic and central inflammatory responses, leading to POCD. However, the relationship between the inflammation and cognitive dysfunction after CRS-HIPEC has not yet been determined.

Therefore, we performed a prospective pilot study to measure the serum levels of inflammatory markers and short-term cognitive function during the perioperative period and to determine whether the systemic inflammation induced by this combined treatment is associated with the impaired cognitive function in patients undergoing CRS-HIPEC. We hypothesized that the increased perioperative systemic inflammation in these patients will be associated with the development of short-term POCD.

## 2. Materials and Methods

This study has been approved by the research ethics committee of Zhongnan Hospital of Wuhan University. With written informed consent, we performed a prospective observational study, which was registered at http://ClinicalTrials.govNCT02462564.

### 2.1. Study Population

**Figure 1:**
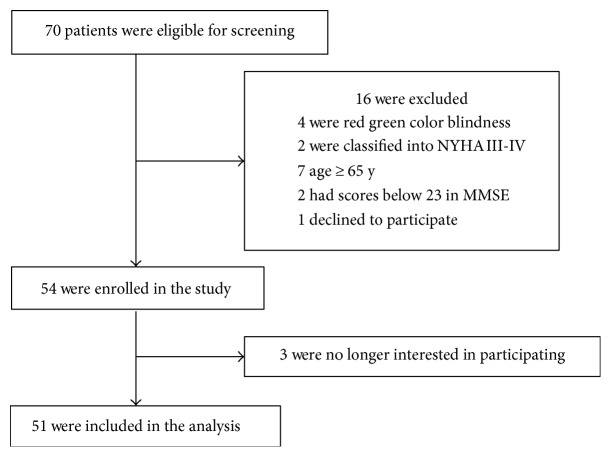
A flow diagram. The flow diagram shows that 70 participants were initially screened for the study and finally, 51 participants were included in the data analysis.

The study took place at Zhongnan Hospital of Wuhan University (Wuhan, China) between June 2014 and May 2015. Eligible patients were aged between 18 to 65 years, scheduled to have CRS-HIPEC under general anesthesia. A total of 70 adults were included in the study (Figure 1, the flow diagram). After reviewing patient medical records, patients were excluded if they had (1) a past medical history of neurological diseases, (2) American Society of Anesthesiologists (ASA) score greater than 4, (3) Mini-Mental State Examination (MMSE) score below 23, (4) a preoperative cardiovascular disease (defined as New York Heart Association III-IV), (5) severe visual or hearing impairment, and (6) a history of drug dependence or alcohol abuse.

We recruited the patient's spouses as the age-matched control group to adjust for the learning effect subsequent to the repeated neuropsychological tests used in the current study. Control participants and patients were identical regarding the exclusion criteria. Each participant underwent the same neuropsychological test battery on 1 w before and 7 d postoperatively.

### 2.2. Surgery and Anesthesia

All patients had exploratory laparotomy, CRS, and HIPEC under general anesthesia and hemodynamic monitoring using the methods described in the previous study [14], by one surgery team to rule out the potential influence of different surgical practice. Briefly, a longitudinal midline incision was made from the xiphoid to the pubic. After that, the peritoneal carcinomatosis was evaluated in detail. Then, maximal CRS was conducted, including the resection of the primary tumor with acceptable margins, any involved adjacent structures, lymphadenectomy, and peritoneotomies [15]. After CRS, 12 L of heated saline containing 30 mg of mitomycin C and 20 mg of hydroxycamptothecin was used to perform HIPEC. The temperature of the perfusion solution was 43.0 ± 0.5°C, and the total HIPEC procedure time was 60 to 90 min. The wound was closed with a relaxation suture, and patient was sent to the intensive care unit (ICU).

General anesthesia was performed by anesthesiologists, who are familiar with the CRS-HIPEC procedure. Electrocardiography, pulse oximetry, capnography, central venous pressure (CVP), and invasive arterial pressure were continuously monitored during anesthesia. General anesthesia was induced with midazolam 0.05–0.1 mg/kg, sufentanil 0.5–1 *μ*g/kg, propofol 1.5–2.5 mg/kg, and rocuronium 0.8–1.0 mg/kg. Then it was maintained with propofol 4 mg/kg/h, remifentanil 0.2 *μ*g/kg/min, cisatracurium 2.0 *μ*g/kg/min, dexmedetomidine 0.5 *μ*g/kg/h, and inhaled sevoflurane 0–4%, to keep bispectral index (BIS) between 40–60. PaCO_2_ was maintained between 35–45 mmHg by mechanical ventilation. Nasopharyngeal temperature was measured, and warming blankets and fluid warming sets were applied during the operation. Ten minutes before and during the HIPEC, all warming equipments were turned off and ice packs were put on both sides of neck, axillae, and groin, in order to prevent hyperthermia. CVP can help to guide the rate of infusion, and we kept mean arterial pressure above 60 mmHg and within 20% of baseline values by the administration of bolus doses of ephedrine (6–12 mg). All patients received a standard postoperative pain management (patient controlled intravenous analgesia: dezocine 0.4 mg/kg and sufentanil 2.0 *μ*g/kg). There were no major complications among the participants during the postoperative period.

### 2.3. Cognitive Function Measurement

Neuropsychological tests were administered 1 w before and 7 d after CRS-HIPEC by a clinical psychologist with Chinese version of the letter-digit coding (LDC) test [16], the Stroop color word test (SCWT) [17], the concept shifting task (CST) test [18], and the visual verbal learning (VVL) test [19]. POCD was diagnosed according to the methodology of the International Study of Postoperative Cognitive Dysfunction (ISPOCD) studies [2].

We collected the normative data from the age-matched control group [1] and assessed the changes in test performance from the preoperative baseline. In control group, the mean and standard deviations (SD) of these differences were calculated. The mean change stands for estimated learning effects. For the patients underwent CRS-HIPEC, we compared baseline values with the 7 d postoperatively test scores, subtracted the average learning effect from the changes, and divided the result by the SD of the performance changes in control groups to get a Z score. The composite Z score was calculated as the sum of the 4 Z scores and normalized with the SD for that sum in the controls. A large positive Z score suggested postoperative cognitive decline. POCD was defined as a composite Z score ≥ 1.96 or a Z score ≥ 1.96 in at least 2 of the 4 subtests [1]. A visual analog scale (VAS) score of 0 to 10 (lower score indicating lower level of pain [20]) was used to assess postoperative pain in the patients 1 d after surgery.

### 2.4. Sample Collection and Measurement

Central venous blood were collected before induction of anesthesia (baseline), at 30 min after the CRS (T_1_), 30 min after the HIPEC (T_2_), and 1 (T_3_) and 24 h (T_4_) after surgery and used to derive serum samples (5 mL per subject) by centrifugation at 1000*g* for 20 min at room temperature. The serum samples were stored at −80°C before usage. The serum levels of IL-1*β*, TNF-*α*, high mobility group box-1 protein (HMGB-1), serum amyloid A (SAA), and S100 calcium-binding protein B (S-100*β*) were measured using enzyme-linked immunosorbent assay (Elabscience Biotechnology Co. Ltd), following the manufacturer's instructions. The detection levels of inflammatory markers and S-100*β* in the assays were 4.688 pg/mL for IL-1*β*, 4.688 pg/mL for TNF-*α*, 18.75 pg/mL for HMGB-1, 0.75 ng/mL for SAA, and 18.75 pg/mL for S-100*β*.

### 2.5. Statistic Analysis

No power calculations were performed, given that this was a pilot study. We recruited almost all the patients in the inclusion year, which we estimated to be 50. The data were expressed as the mean ± standard error of mean (SEM), the median and interquartile range (IQR, 25–75% percentile), or count (%). POCD incidence was presented as a percentage. The Kolmogorov-Smirnov method was used first to test the normality of all of the variables, and we found the data were approximately normally distributed. We used unpaired and paired *t*-tests to compare numerical variables, and chi-squared test was applied to analyze categorical variables. Differences in IL-1*β*, TNF-*α*, HMGB-1, SAA, and S-100*β* levels over time and between patients with or without POCD were compared by two-way repeated measure analysis of variance with Bonferroni correction. We also performed a logistic regression to model the binary outcome of POCD to determine the association between the maximum concentrations of inflammatory markers or S-100*β* in serum and POCD. *P* values less than 0.05 were considered statistically significant. SPSS statistical software, version 21.0 (SPSS Inc, Chicago, IL) and GraphPad Prism software, version 6.01 (GraphPad Software Inc, La Jolla, CA) were used for data analysis.

## 3. Results

### 3.1. Participant Characteristics

Seventy eligible patients were screened, 54 of whom gave the written informed consent. Three patients were excluded from the study. Reasons for dropout are shown in Figure 1. Therefore, 51 patients (*n* = 51) remained for analysis.

The characteristics and baseline cognitive performances were similar in patients and control subjects before CRS-HIPEC (Table 1).  Table 2 demonstrates the raw scores of cognitive tests in patients. According to the POCD diagnostic criteria, twenty patients (39.2%) developed POCD 1 w after surgery.

Table 3 shows the demographic and clinical data of the patients. Patients who developed POCD were older (median [IQR]; 63 [56.3–65] versus 44 [30–51] years, *P* < 0.001) and had higher fasting blood sugar (FBS) (8.85 [7.68–7.98] versus 6.20 [4.90–6.60] mmol/L, *P* = 0.006) than patients without POCD. Seventeen patients (85%) in POCD group and 13 patients (41.9%) in non-POCD group were female (*P* = 0.032). More patients with POCD had a history of hypertension (60% versus 16.1%, *P* = 0.021). There were 5 diabetics (25%) in POCD group, while no patient in non-POCD group had diabetes. More patients (60% versus 16.1%, *P* = 0.021) who developed POCD suffered from ovarian cancer than those who did not. No other distinct differences were seen in other demographic variables and clinical characteristics, such as weight, education, ASA classification, VAS scores, and the surgical and anesthesia parameters between patients with and without POCD (*P* > 0.05). During HIPEC, 13 patients (65%) in POCD group and 21 patients (67.7%) in non-POCD group, core temperature reached levels greater than 37.5°C, although the cooling measures had been taken. Additionally, no significant differences were observed in core temperature between the patients with and without POCD (*P* > 0.05).

### 3.2. Perioperative Levels of Inflammatory Markers and S-100*β* in Serum and POCD

We examined the concentrations of inflammatory markers, such as IL-1*β*, TNF-*α*, HMGB-1, and SAA, as well as S-100*β* before induction of anesthesia (baseline), at 30 min after the CRS (T_1_), 30 min after the HIPEC (T_2_), and 1 (T_3_) and 24 h (T_4_) after surgery. There were 7 missing values in the inflammatory markers (6 for IL-1*β* and 1 for SAA), due to the measurements under the detection limits.

**Figure 2:**
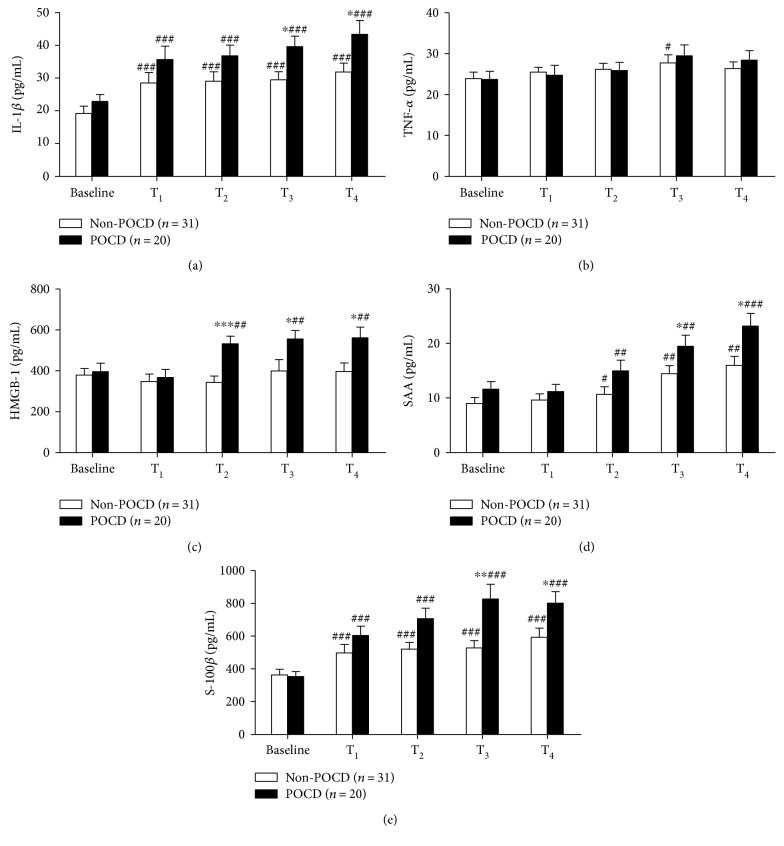
Analysis of inflammatory markers, such as interleukin 1*β* (IL-1*β*) (a), tumor necrosis factor *α* (TNF-*α*) (b), high mobility group box-1 protein (HMGB-1) (c), serum amyloid A (SAA) (d), and S100 calcium-binding protein B (S-100*β*) (e) in patients with and without postoperative cognitive dysfunction (POCD). Data are presented as mean ± standard error of mean (SEM). ^#^*P* < 0.05 versus baseline value; ^##^*P* < 0.01 versus baseline value; ^###^*P* < 0.001 versus baseline value; ^∗^*P* < 0.05 versus non-POCD group; ^∗∗^*P* < 0.01 versus non-POCD group; and ^∗∗∗^*P* < 0.001 versus non-POCD group.

We found that IL-1*β* level at all the time point was higher than the baseline in both POCD group (*P* < 0.001) and non-POCD group (*P* < 0.001). A significant difference between patients with and without POCD was observed at 1 and 24 h after CRS-HIPEC (39.57 ± 3.19 versus 29.49 ± 2.49 pg/mL *P* = 0.015 and 43.33 ± 4.23 versus 31.80 ± 2.78 pg/mL, *P* = 0.021, resp.) (Figure 2(a)).

The changes of TNF-*α* concentration were just observed at T_3_, compared to the baseline value in patients without POCD (27.76 ± 1.96 versus 23.88 ± 1.59 pg/mL, *P* = 0.049) but not observed at other time point, compared to the baseline value in both groups (*P* > 0.05 and *P* > 0.05). The concentration of TNF-*α* did not differ between the patients who developed cognitive dysfunction after surgery and those who did not (*P* > 0.05) (Figure 2(b)).

The serum level of HMGB-1 was higher than baseline value in the POCD group at T_2_, T_3_, and T_4_ (*P* = 0.002, *P* = 0.006, and *P* = 0.001, resp.). In addition, HMGB-1 level in serum was higher in patients with POCD than that in patients without POCD at T_2_, T_3_, and T_4_ (532.83 ± 36.32 versus 343.54 ± 30.98 pg/mL, *P* < 0.001, 555.72 ± 42.17 versus 399.50 ± 55.27 pg/mL, *P* = 0.047, and 561.94 ± 52.14 versus 396.36 ± 42.15 pg/mL, *P* = 0.017, resp.) (Figure 2(c)).

In both groups, SAA level at T_2_, T_3_, and T_4_ was higher than the baseline value (POCD group: *P* = 0.001, *P* = 0.003, and *P* < 0.001, respectively; non-POCD group: *P* = 0.040, *P* = 0.004, and *P* = 0.001, resp.). Patients who developed cognitive dysfunction after surgery had higher SAA level at T_3_ and T_4_, compared with those who did not (19.47 ± 2.04 versus 14.46 ± 1.49 ng/mL, *P* = 0.049 and 23.19 ± 2.33 versus 15.99 ± 1.63 ng/mL, *P* = 0.012, resp.) (Figure 2(d)).

The concentration of S-100*β* was increased at all the perioperative time point in patients with or without POCD, compared to baseline value (POCD group: *P* < 0.001; non-POCD group: *P* < 0.001). There were significant differences in the serum concentration of S-100*β* at T_3_ and T_4_ between the patients who developed POCD and those did not (827.34 ± 89.16 versus 527.56 ± 45.75 pg/mL, *P* = 0.002 and 802.11 ± 69.75 versus 593.65 ± 55.75 pg/mL, *P* = 0.025, resp.) (Figure 2(e)).

According to logistic regression analyses, the odds ratio (OR) estimates for serum levels of inflammatory markers and S-100*β* on POCD are showed in Table 4. The peak levels of IL-1*β*, HMGB-1, and SAA in serum occurred 24 h after CRS-HIPEC (T_4_), and serum S-100*β* level reached the maximum value 1 h after surgery (T_3_). There was an association between POCD and the maximum IL-1*β* (T_4_) and S-100*β* (T_3_) concentrations in serum, but not the maximum serum HMGB-1 (T_4_) or SAA (T_4_) concentrations. Additionally, when confounding factors such as age and FBS were included in the multivariate model, the OR for IL-1*β* increased modestly from 1.26 to 1.41 and the OR for S-100*β* increased from 1.13 to 1.25, respectively.

## 4. Discussion

In this pilot study, we found that POCD was common in patients undergoing CRS-HIPEC. Impairments in cognitive function occurred in 20 (39.2%) of the 51 patients. Additionally, serum concentrations of IL-1*β*, HMGB-1, and SAA, as well as S-100*β*, increased during and after CRS-HIPEC. More importantly, elevated levels of IL-1*β* and S-100*β* were associated with cognitive decline 1 w after surgery. Taken together, these findings indicate that the increased systemic inflammation is associated with the occurrence of cognitive impairments after CRS-HIPEC.

In the present study, POCD was assessed according to the study of Hansen et al. [21], and the methodology from ISPOCD [2]. Moller et al. [1] found that POCD was present in 25.8% of elder patients 1 w after major noncardiac surgery. In our studies, we found that the incidence of POCD was 39.2%, 7 d after the CRS-HIPEC, which was higher than early POCD incidence found by ISPOCD group [1]. It was known that more complicated surgery was more likely to exhibit POCD at hospital discharge. Thus, the higher POCD incidence in the current study may be related to the type of surgery, since CRS-HIPEC is a complicated surgery. However, such discrepancies could also originate from the different intervals between testing sessions and exclusion criteria on preoperative MMSE, as well as the small sample size in our studies, pending further investigations.

Age has been implicated as a risk factor and predictor of early POCD [1, 3]. In the present study, the patients who developed short-term POCD after the CRS-HIPEC were older than those did not. This finding is consistent with the results from Newman et al. [3] and ISPOCD1 study [1]. It is unclear why the patients with ovarian cancer tended to develop cognitive impairments after surgery. One possible explanation for this finding might be that the extent of resection for the CRS between the patients who suffered fromovarian cancer and gastrointestinal cancer was different. However, in our study, patients with or without POCD had statistically similar blood loss, surgery time, and transfusion volume. Additionally, the sex differences between the POCD and non-POCD group may be related to the primary causes (ovarian cancer versus gastrointestinal tumor) of the patients. Furthermore, in the current study, more patients with POCD had a history of hypertension or diabetes. Diabetes mellitus (DM) type 2 is associated with accelerated cognitive decline and structural brain abnormalities [22]. Likewise, hypertension has been reported as a major risk factor for stroke and dementia and is associated with MRI evidence of white matter hyperintensity (WMH) and reduced brain volumes [23]. More importantly, the neuronal loss was found in the hippocampus when diabetes and hypertension are combined [14]. These findings indicate that patients with a history of hypertension or diabetes may have some special underlying changes in the vulnerable brain that facilitate the occurrence of cognitive dysfunction after CRS-HIPEC, although diabetes has not been identified as an independent risk factor for POCD [24]. We also noticed that patients who exhibited cognitive impairment after CRS-HIPEC had higher perioperative FBS. It is possible that the higher blood sugar reflected more severe inflammatory response in patients with POCD, since the release of cytokines subsequent to the surgical trauma plays a role in the regulation of postoperative insulin resistance [25].

The relationship between acute postoperative pain and POCD has been reported [26]. In our study, all patients had a standard postoperative pain control and there were no differences in VAS scores between both groups 1 d postoperatively, suggesting that the postoperative pain might not contribute to the cognitive function differences.

During HIPEC, the high-temperature (43.0 ± 0.5°C) perfusion solution was used. However, the core temperature did not differ between patients with and without POCD. Few studies have showed the relationship between the hyperthermia and POCD. However, some studies have indicated the association between slower rewarming during cardiac surgery and better postoperative cognitive outcome [27]. It is possible that the brain could be susceptible to insult during rewarming from hypothermia, in particular when the cerebral autoregulation mechanisms cannot compensate for a sudden metabolic activity increase subsequent to the changes in temperature [28]. In the current study and the study from Miao et al. [28], the changes of core temperature were short-lived and mild. Therefore, these changes do not appear to contribute to postoperative cognitive impairments.

Inflammatory response plays a crucial role in the development of POCD. Surgical trauma engages the innate immune system to release proinflammatory cytokines, in particular IL-1*β* and TNF-*α*. These cytokine signals can be transmitted to the brain and lead to neuroinflammation through direct neural pathways (via primary autonomic afferents), transport across the blood-brain barrier (BBB), or entry via the disrupted BBB. Increased brain proinflammatory cytokines can overactivate microglia, which induces further cytokine release in the brain and fuels a vicious cycle of neuroinflammation [29, 30]. Furthermore, overactivated microglia create neurotoxic responses, cause neuronal injury, and affect neuronal function, leading to POCD [31, 32]. The intensity of postoperative inflammatory response depends on the incision size and the severity of surgery [11, 12]. CRS-HIPEC had a large incision and a complicated surgical course, reflected by the long duration, dramatic hemodynamic disturbance, for instance, tachycardia and hypotension, and metabolic perturbations, such as deterioration of gas exchange and acidosis during the procedure, although these changes are variable and short-lived [28]. Therefore, in the current study, the increased levels of inflammatory markers during and after CRS-HIPEC, as well as the association between elevated levels of inflammatory markers and cognitive decline 1 w after surgery, support our assumption that inflammatory response plays an important role in the development of POCD following CRS-HIPEC.

Cytokines, such as IL-1*β* and TNF-*α*, have been reported to play a pivotal role in surgery-induced inflammation and cognitive dysfunction [4, 6]. In the current study, we found that the patients who developed POCD had a higher serum level of IL-1*β* level at 1 and 24 h after CRS-HIPEC than the patients without POCD. We also found the association between POCD and the maximum IL-1*β* concentration in serum. These findings indicate that enhanced level of IL-1*β* postoperatively is associated with the occurrence of POCD. In the present study, the subtle changes of TNF-*α* concentration were only observed at 1 h after surgery in patients without POCD, and there were no significant differences in TNF-*α* concentration between the patients with and without POCD. These results are consistent with a recent study, finding that serum TNF-*α* concentration did not increase at the end of abdominal surgery and on 1, 2, and 3 d postoperatively [33]. In our study, we did not detect an obvious change in TNF-*α* level associated with an increase in IL-1*β*. The discrepancy might derive from the different time points and type of surgery. The study of Terrando et al. showed that TNF-*α* was the first cytokine to be released, compared with other inflammatory cytokines such as IL-1*β* and IL-6, following orthopedic surgery and peaked at 30 min after surgery in adult mice [6]. Of note is that, in this pilot study, we did not measure serum TNF-*α* level at this time point. Besides, the small sample size might result in an inconsistency, and it requires further investigations.

HMGB-1 is a late-phase cytokine, which is released extracellularly in response to systemic inflammation caused by infection, shock, or trauma [34]. Recently, HMGB-1 has been implicated as a crucial inflammatory mediator in surgical stress [35]. Increasing evidence also suggests the relationship between elevated HMGB-1 level and impaired cognitive function after surgery [36]. For example, Lin et al. [37] reported that serum HMGB-1 increases significantly after major gastrointestinal surgery in elderly patients and such elevation is associated with the occurrence of POCD. Our results showed that serum HMGB-1 level of both groups increased gradually, reaching the peak at 24 h after CRS-HIPEC, and patients with POCD had significantly higher serum HMGB-1 level at 30 min after HIPEC, as well as 1 and 24 h after surgery compared to the patients without POCD. These findings are in accordance with previous study [37]. Interestingly, the association between maximum serum HMGB-1 concentration and POCD was not observed in the logistic regression analyses. This is probably due to the relatively small sample size in our study.

S-100*β* has been identified as a biomarker of BBB disruption in neurodegenerative diseases and traumatic brain injury (TBI). The changes in BBB permeability after surgical trauma have been found in aged POCD rats [38]. Terrando et al. [32] also found that surgical trauma engages the innate immune system through nuclear factor- (NF-) *κ*B-dependent signaling to activate proinflammatory cytokines that changes BBB permeability. Therefore, in our study, both the association between POCD and serum maximum S-100*β* concentration and the elevated level of S-100*β* in the patients who developed cognitive decline after surgery may indicate the changes of BBB permeability after CRS-HIPEC, which was induced by the proinflammatory cytokines.

Accumulating evidence suggests that the acute-phase protein SAA plays an active role in inflammation. SAA is produced when mammals sense potentially harmful environmental cues, including trauma, infection, tumor growth, surgery, and severe stress [39]. Published studies suggest that SAA may have profound effects on innate immunity as a result of its chemotactic and cytokine-inducing activities [40]. On the other hand, SAA may be related to cognitive dysfunction. Trollor et al. [41] found that there is an association between high SAA level and lower performance on processing speed and fine motor domains in elderly participants. We measured the perioperative SAA in patients undergoing CRS-HIPEC, given that enhanced SAA might be related to inflammatory responses and cognitive decline and found that patients who exhibited cognitive impairment after CRS-HIPEC had a higher SAA level at 1 and 24 h after surgery.

There are some potential limitations of our study. First, the current pilot study was not sufficiently powered to provide strong evidence for the incidence of cognitive decline after CRS-HIPEC and the role of systemic inflammation in the impairments of cognitive function after this combined surgery, due to the small sample size. A larger study with adequate power is indicated to validate our results. Second, in our study, we just focused on four time points. These time points could not cover the time course of inflammatory cytokine expressions in detail in patients undergoing CRS-HIPEC. Further investigations are needed on this point. Third, patients with delirium have difficulty on attention, concentrating, and distractibility and have been shown to score lower on neuropsychological tests [42]. We did not measure delirium in our study. Accordingly, we might have overestimated the incidence of short-term POCD in patients undergoing CRS-HIPEC, given the facts that we could not distinguish the patients with postoperative delirium from the patients with POCD. Delirium diagnosis tools, such as the confusion assessment method (CAM), should be used in the future studies. Finally, it is well known that depression and anxiety are associated with cognitive deterioration and may have a negative impact on motivation and ability to complete neuropsychological testing [43]. We did not evaluate the changes in mood in the current study, although we chose 1 w before operation as the time point to test the baseline cognitive function to avoid the influence of the preoperative anxiety and depression.

In conclusion, in the pilot study, we found that patients who undergo CRS-HIPEC are at increased risk of developing POCD, which might be related to the increased systemic inflammatory response induced by this complicated surgery. The results suggest the need for a larger RCT to replicate and confirm these findings, as well as explore approaches that attenuate the inflammatory response and improve POCD after complicated surgery. Design considerations for such a trial should include the need for multiple sites, longer inclusion periods, as well as more types of surgery to facilitate adequate recruitment, and extension of follow-up periods.

## Figures and Tables

**Table 1:** Demographics and baseline cognitive performances in patients and controls.

Variable	Patients (*n* = 51)	Controls (*n* = 20)	*P* value
Age (median, [IQR]; y)	51 (38–62)	50 (37–59)	0.203
Male, *n* (%)	21 (41.1)	8 (40)	0.928
Weight (median, [IQR]; kg)	60 (50–65)	57 (51–64)	0.196
Education (median, [IQR]; y)	15 (12–15)	15 (12–15)	0.557
LDC (median, [IQR]; points)	38 (28–56)	34 (27–58)	0.276
SCWT (median, [IQR]; points)	36 (29–48)	38 (29–42)	0.194
CST (median, [IQR]; points)	69 (47–100)	69 (51–100)	0.333
VVL (median, [IQR]; points)	10 (9–12)	11 (10–12)	0.387

Data are presented as mean (IQR) or count (%).

**Table 2:** The raw scores of cognitive tests in patients.

	Baseline	7 days after the surgery
LDC (median, [IQR]; points)	38 (28–56)	37 (20–55)
SCWT (median, [IQR]; points)	36 (29–48)	34 (22–50)
CST (median, [IQR]; points)	69 (47–100)	79 (42–154)
VVL (median, [IQR]; points)	10 (9–12)	12 (9–14)

Data are presented as mean (IQR). The higher scores suggest better cognitive function except CST.

**Table 3:** Demographic and clinical characteristics of patients with and without POCD.

	Patients with POCD (*n* = 20)	Patients without POCD (*n* = 31)	*P* value
Demographics			
Age (median, [IQR]; y)	63 (56.3–65)	44 (30–51)	0.000^∗∗^
Female, *n* (%)	17 (85)	13 (41.9)	0.032^∗^
Weight (median, [IQR]; kg)	64.5 (50–65)	60 (51–68)	0.591
Education (median, [IQR]; y)	12 (12–15)	15 (12–15)	0.426
Medical history (*n* [%])			
Hypertension	12 (60)	5 (16.1)	0.021^∗^
Coronary disease	3 (15)	0 (0)	0.142
Diabetes	5 (25)	0 (0)	0.016^∗^
Ovarian cancer	12 (60)	5 (16.1)	0.021^∗^
Perioperative factors			
ASA class I-II, *n* (%)	17 (85)	31 (100)	0.142
Inhalation anesthesia			
Duration (median, [IQR]; min)	390 (307.5–550)	500 (375–650)	0.312
>1.5MAC, *n* (%)	5 (25)	16 (51.6)	0.158
Surgery			
Duration (median, [IQR]; min)	505 (345–597.5)	565 (375–650)	0.557
HIPEC duration (median, [IQR]; min)	60 (60–112.5)	60 (60–90)	0.548
Hypotension (median, [IQR]; frequency)	3 (2–8)	2 (0–4)	0.137
Hyperthermia, *n* (%)	13 (65)	21 (67.7)	1.000
Transfusion volume (median, [IQR]; mL/h)	646.2 (552–737.5)	640 (553.8–736)	0.872
Urine volume (median, [IQR]; mL/h)	176.8 (152.4–365)	184.6 (103.4–231.6)	0.167
Blood loss (median, [IQR]; mL)	600 (425–1150)	600 (500–800)	0.614
Pain			
VAS (median, [IQR]; points)	5 (2.6–6.8)	3 (2–4)	0.125
Arterial blood gas analysis			
PH (median, [IQR])			0.800
Baseline	7.45 (7.43–7.49)	7.47 (7.40–7.50)	
T_1_	7.45 (7.41–7.50)	7.44 (7.40–7.50)	
T_2_	7.42 (7.38–7.43)	7.42 (7.38–7.45)	
PaCO_2_ (median, [IQR]; mmHg)			0.197
Baseline	26.70 (24.10–30.78)	29.90 (26.00–33.70)	
T_1_	26.40 (23.55–28.85)	28.20 (25.10–32.90)	
T_2_	30.40 (28.18–31.30)	30.20 (27.50–35.00)	
Hb (median, [IQR]; g/dL)			0.292
Baseline	10.65 (9.28–11.90)	11.30 (10.70–12.20)	
T_1_	9.75 (8.78–11.53)	10.80 (10.10–12.00)	
T_2_	9.95 (8.83–12.10)	10.90 (9.70–11.90)	
FBS (median, [IQR]; mmol/L)			0.006^∗∗^
Baseline	4.95 (4.00–6.53)	3.50 (3.00–4.70)	
T_1_	7.00 (5.45–8.20)	5.20 (4.30–6.10)	
T_2_	8.85 (7.68–9.68)	6.20 (4.90–6.60)	

Data are presented as mean (IQR) or count (%). The length of inhalation anesthesia was defined as the duration of sevoflurane exposure; patients depend on more than 1.5 MAC sevoflurane inhalation to maintain BIS between 40 and 60 during the operation were classified into “>1.5 MAC;” the length of surgery was defined from the time of initial incision to the time of closure of the skin; hypotension was defined as 30% under basal blood pressure; hyperthermia was defined as patients' body temperature was higher than 37.5°C during the operation; arterial blood gas analysis was carried out at three time points (T_0_, before induction of anesthesia; T_1_, 30 min after the CRS; and T_2_, 30 min after the HIPEC); ^∗^*P* < 0.05 and ^∗∗^*P* < 0.01; variables that were statistically significant differences between the POCD and non-POCD groups included age, sex, hypertension, diabetes, primary causes, and FBS.

**Table 4:** OR estimates for serum levels of inflammatory markers and S-100*β* on POCD from logistic regression analyses.

	Unadjusted		Adjusted by age and FBS	
	OR	95% CI	OR	95% CI
Maximum IL-1*β* concentration^a^	1.26	(1.09, 1.55)	1.41	(1.08, 1.85)
Maximum HMGB-1 concentration^a^	1.09	(0.94, 1.46)	1.02	(0.91, 1.38)
Maximum SAA concentration^a^	1.01	(0.81, 1.29)	0.98	(0.75, 1.24)
Maximum S-100*β* concentration^b^	1.15	(1.07, 1.44)	1.25	(1.09, 1.63)

OR: odds ratio; CI: confidence interval; FBS: fasting blood sugar; IL-1*β*: interleukin 1*β*; HMGB-1: high mobility group box-1 protein; SAA: serum amyloid A; S-100*β*: S100 calcium-binding protein B. ORs for ^a^24 h postoperatively, ORs for ^b^1 h postoperatively.
